# Functional Implications of Dale's Law in Balanced Neuronal Network Dynamics and Decision Making

**DOI:** 10.3389/fnins.2022.801847

**Published:** 2022-02-28

**Authors:** Victor J. Barranca, Asha Bhuiyan, Max Sundgren, Fangzhou Xing

**Affiliations:** Department of Mathematics and Statistics, Swarthmore College, Swarthmore, PA, United States

**Keywords:** neuronal networks, decision making, balanced networks, nonlinear dynamics, Dale's law

## Abstract

The notion that a neuron transmits the same set of neurotransmitters at all of its post-synaptic connections, typically known as Dale's law, is well supported throughout the majority of the brain and is assumed in almost all theoretical studies investigating the mechanisms for computation in neuronal networks. Dale's law has numerous functional implications in fundamental sensory processing and decision-making tasks, and it plays a key role in the current understanding of the structure-function relationship in the brain. However, since exceptions to Dale's law have been discovered for certain neurons and because other biological systems with complex network structure incorporate individual units that send both positive and negative feedback signals, we investigate the functional implications of network model dynamics that violate Dale's law by allowing each neuron to send out both excitatory and inhibitory signals to its neighbors. We show how balanced network dynamics, in which large excitatory and inhibitory inputs are dynamically adjusted such that input fluctuations produce irregular firing events, are theoretically preserved for a single population of neurons violating Dale's law. We further leverage this single-population network model in the context of two competing pools of neurons to demonstrate that effective decision-making dynamics are also produced, agreeing with experimental observations from honeybee dynamics in selecting a food source and artificial neural networks trained in optimal selection. Through direct comparison with the classical two-population balanced neuronal network, we argue that the one-population network demonstrates more robust balanced activity for systems with less computational units, such as honeybee colonies, whereas the two-population network exhibits a more rapid response to temporal variations in network inputs, as required by the brain. We expect this study will shed light on the role of neurons violating Dale's law found in experiment as well as shared design principles across biological systems that perform complex computations.

## 1. Introduction

Computation in the brain is closely tied to the complex network architecture of neuronal interactions and the host of electrochemical signals transmitted between neurons (Boccaletti et al., 2006; Stevenson et al., 2008; Gomez-Rodriguez et al., 2012). Studies relating network connectivity to biological function have revealed potential mechanisms for fundamental cognitive processes, characterizing important aspects of stimulus encoding in perception and evidence integration in choice (Hubel and Wiesel, 1972; Shadlen and Newsome, 2001; Hafting et al., 2005; Rangan et al., 2005; Bassett et al., 2010; Vzquez-Rodrguez et al., 2019). The network adjacency matrix dictates the general interaction structure for neuronal networks, revealing certain dynamical properties such as synchronizability and functional modules (Newman, 2003; Arenas et al., 2006; Estrada and Higham, 2010; Sporns, 2014; Barranca et al., 2015), but neurons also release diverse neurotransmitters that affect their neighbors in unique ways and this necessitates a more detailed portrait of neuronal communication beyond the binary adjacency matrix.

One classical organizing feature for neuronal interactions supported by strong experimental evidence is *Dale's law*, which typically states that a neuron releases the same types of neurotransmitters at all of its post-synpatic connections regardless of the nature of the post-synaptic neuron (Kandel, 1968; Strata and Harvey, 1999). Theoretical investigations and mathematical models almost unanimously reflect Dale's law by separating a neuronal network into two disjoint populations of neurons based on the collective action of their neurotransmitters, where one population is composed of excitatory neurons that upon firing increase the probability of their neighbors firing and the other population is composed of inhibitory neurons that analogously decrease the firing probability of their neighbors (Orlandi et al., 2014; Barranca and Zhou, 2019; Lian et al., 2019). However, experiments demonstrate that some neurons indeed violate Dale's law, for example through state-dependent co-transmission or the release of different neurotransmitters at distinct release sites (Jonas et al., 1998; Nicoll and Malenka, 1998; Ludwig and Leng, 2006; Svensson et al., 2018). The functional impact of such violations to Dale's law in the brain remains largely unexplored.

In a similar vein, biological systems, such as colonies of honeybees, also communicate *via* explicit excitatory and inhibitory feedback signals (Couzin, 2009; Marshall et al., 2009; Seeley et al., 2012). In collectively selecting a food source akin to decision making among neurons in the brain, each bee lacks knowledge of the detailed state of the colony, yet the entire group is able to effectively coordinate choices when accumulated positive feedback passes a threshold. Honeybees use waggle dances to encourage additional bees to forage at a profitable food source and also utilize stop signals to discourage other bees from foraging at a sub-optimal location (Seeley et al., 2012; Von Frisch, 2013; Borofsky et al., 2020). In contrast to the classical notion of Dale's law, a single honeybee can send out either type of feedback signal depending on its circumstances. In engineered systems, such as artificial neural networks, each unit generally has the potential to increase the activity of some of its neighbors while decreasing the activity of others, and this feature is typically preserved even upon sufficient learning for optimal task performance (Wang and Yeung, 2013; Deng et al., 2014; Chan et al., 2015; Schmidhuber, 2015; Barranca, 2021). Why do neuronal networks largely follow Dale's law unlike other systems and what classical brain computations are preserved by neuronal networks that violate it?

To address this key question, we formulate and analyze a mechanistic network model composed of a single population of neurons that can each transmit both excitatory and inhibitory signals. Like physiological neuronal networks, we demonstrate that this single-population model network exhibits asynchronous and irregular dynamics. In doing so, we provide a new generalization for the theory of balanced network dynamics (van Vreeswijk and Sompolinsky, 1996; Troyer and Miller, 1997; Vogels and Abbott, 2005), which posits that strong excitatory and inhibitory inputs into a given neuron are dynamically balanced in time such that infrequent input fluctuations result in the irregular activity typically observed *in vivo* (Haider et al., 2006; Miura et al., 2007; London et al., 2010). Such balanced dynamics are hypothesized to have numerous functional advantages, such as fast input tracking (van Vreeswijk and Sompolinsky, 1996), robust spatial working memory (Lim and Goldman, 2014), efficient predictive coding (Boerlin et al., 2013), and effective pattern learning (Ingrosso and Abbott, 2019). We show that the single-population model exhibits a higher degree of balance than the classical two-population model obeying Dale's law for relatively small network sizes, giving insight into why honeybee colonies and other smaller-scale systems may violate Dale's law. While we establish that the one-population model well replicates the rapid response to time-varying inputs found for classical balanced networks, we observe that the two-population network is able to yet more quickly respond and we conjecture this may explain the ubiquity of Dale's law in neuronal networks that require fast computations for survival. In comparing the computational properties of the two distinct network models, we further demonstrate that the single-population model fosters successful decision-making dynamics for two-alternative tasks, thereby carrying out a computation that is fundamental in numerous biological and engineered systems. We hypothesize that networks violating Dale's law are capable of more flexible computations, particularly for relatively small systems, and that functional or energetic constraints in large-scale systems may have promoted Dale's law among neurons.

The remainder of the paper is organized as follows. In Section 2.1, we briefly summarize the key aspects of balanced dynamics in the context of a two-population network of excitatory and inhibitory neurons, and then in Section 2.2, we formulate our one-population network model that violates Dale's law. We demonstrate that the one-population model exhibits robust balanced network dynamics, both theoretically and numerically, in Section 3.1 and compare how the balanced dynamics for the two different network models scale with network size in Section 3.2. The input-response properties for the network models are contrasted in Section 3.3, and we further show that the one-population model facilitates successful decision-making dynamics analogous to classical balanced networks in Section 3.4. Finally, we reflect on our findings and potential directions for future investigation in Section 4.

## 2. Models and Methods

### 2.1. Two-Population Model and Balanced Network Dynamics

We first introduce the conventional two-population network model that obeys Dale's law and summarize its dynamical properties in the balanced operating regime; these dynamical characteristics will provide a benchmark for comparison with the activity of the one-population model violating Dale's law that will be introduced in the next section. For concreteness and analytical tractability, we consider networks composed of the binary-state neurons with which balanced network theory was originally studied (van Vreeswijk and Sompolinsky, 1996, 1998). The two-population network is composed of *N* neurons, where *N*_*E*_ neurons are excitatory (E) and *N*_*I*_ neurons are inhibitory (I). The state of the *i*th neuron in the *k*th population (*k* = *E, I*) at time *t* is governed by


(1) $$\begin{array}{l} \sigma_{k}^{i}\left(t\right)=H\left(\mu_{k}^{i}\left(t\right)-\theta_{k}\right), \end{array}$$

where θ_*k*_ is the firing threshold for the neurons in population *k* and *H*(·) is the Heaviside function. Each neuron, therefore, has two possible states, namely firing (σ = 1) or subthreshold (σ = 0), depending on whether its total input reaches a firing threshold. The total drive $\mu_{k}^{i}\left(t\right)$ into the *i*th neuron in the *k*th population at time *t* is


(2) $$\begin{array}{l} \mu_{k}^{i}\left(t\right)=\overset{N_{E}}{\underset{j=1}{\sum }}J_{kE}^{ij}\sigma_{E}^{j}\left(t\right)+\overset{N_{I}}{\underset{j=1}{\sum }}J_{kI}^{ij}\sigma_{I}^{j}\left(t\right)+\mu_{k}^{0}, \end{array}$$

where $J_{kl}^{ij}$ denotes the connection strength between the *i*th post-synaptic neuron in the *k*th population and the *j*th pre-synaptic neuron in the *l*th population (*l* = *E, I*), and $\mu_{k}^{0}$ is the strength of the external input into a neuron in the *k*th population. The recurrent connection strength $J_{kl}^{ij}$ is chosen to be $J_{kl}/\sqrt{K}$ with probability *K*/*N*_*l*_ and 0 otherwise, abstracting over any detailed network structure to highlight the impact of Dale's law in particular. Hence, a given neuron is expected to receive *K* incoming synaptic connections of each type. Reflecting the nature of the two populations, the excitatory connection strength *J*_*kE*_ is positive and the inhibitory connection strength *J*_*kI*_ is negative. In evolving the state of each neuron, the mean time between subsequent updates is τ_*E*_ = 10 ms for excitatory neurons and τ_*I*_ = 9 ms for inhibitory neurons, based on experimental estimates of cortical membrane potential time constants (McCormick et al., 1985; Shelley et al., 2002).

Since biophysical noise sources are generally unable to account for the irregular neuronal dynamics observed *in vivo* (Softky and Koch, 1993; Faisal et al., 2008), the model requires that asynchronous dynamics are fully produced by the recurrent interactions among neurons. We, therefore, assume constant external inputs $\mu_{E}^{0}=f_{E}m_{0}\sqrt{K}$ and $\mu_{I}^{0}=f_{I}m_{0}\sqrt{K}$, where the positive external input strength is modulated by non-negative and O(1) parameter *m*_0_. It is assumed that *f*_*E*_ as well as *f*_*I*_ are positive O(1) parameters scaling the relative external input into the excitatory and inhibitory populations, respectively. The recurrent connection strengths and firing thresholds are also O(1) parameters, and the relationship between them and the external input scalings primarily determines the network dynamical regime as will be further discussed in Section 3.1.

**Figure 1:**
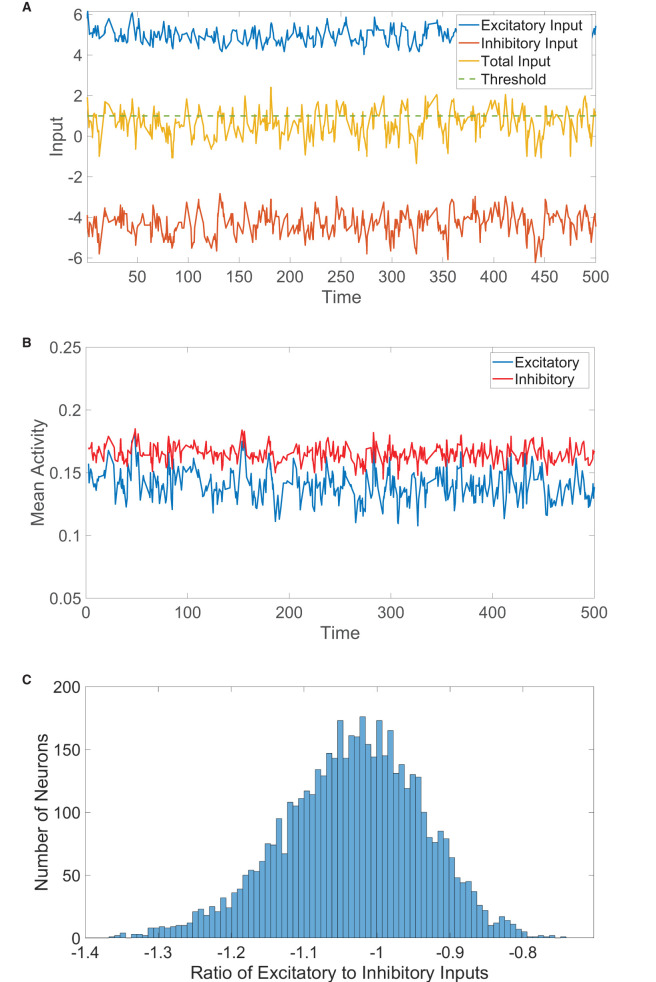
Two-population balanced network dynamics. **(A)** Excitatory (blue), inhibitory (red), and net (yellow) inputs into a sample excitatory neuron in a two-population balanced network. The dashed (green) line indicates the firing threshold. **(B)** The population-averaged state (mean activity) of the excitatory (blue) and inhibitory (red) populations as a function of time. **(C)** Histogram of the ratio between time-averaged excitatory and inhibitory inputs across the two-population neuronal network. Parameters utilized are *J*_*EE*_ = *J*_*IE*_ = 1, *J*_*II*_ = −1.8, *J*_*EI*_ = −2, *N*_*E*_ = 4, 000, *N*_*I*_ = 1, 000, *f*_*E*_ = 1, *f*_*I*_ = 0.8, *m*_0_ = 0.2, *K* = 200, θ_*E*_ = 1, and θ_*I*_ = 0.8.

Considering each neuron is expected to receive *K* excitatory incoming synaptic connections and *K* inhibitory incoming synaptic connections, if the excitatory and inhibitory inputs are not well balanced, the total drive is $O\left(\sqrt{K}\right)$. Since the total drive in this case is larger in magnitude than the firing threshold, each neuron would generally either fire or remain subthreshold for all time, thereby demonstrating highly regular and unrealistic dynamics. When the total drive is instead of the same order as the O(1) firing threshold, intermittent O(1) input fluctuations become largely responsible for the exact timing of firing events and their highly irregular distribution. This theoretically produces balanced network dynamics, whose key features we summarize in Figure 1 and later compare to those produced for the one-population model violating Dale's law.

In Figure 1A, we plot the total excitatory and inhibitory inputs over time for a sample neuron in the balanced state. The two input types are much larger in magnitude than the firing threshold, but the excitation and inhibition dynamically cancel and cause the total input to irregularly exceed threshold. We may similarly represent the resultant dynamics on the network level using the population-averaged state, which we will refer to as the *mean activity* and will serve to approximate the firing rate of each population. The mean activity for the *k*th population at time *t* is thus $m_{k}\left(t\right)=\frac{1}{N_{k}}\sum_{i=1}^{N_{k}}\sigma_{k}^{i}\left(t\right)$. The mean activity for each population is depicted over time in Figure 1B, exhibiting a high degree of statistical stationarity and relatively infrequent firing events. In comparison to the time-averaged mean activity over a long time horizon, *m*_*k*_, we see that fluctuations in the mean activity with time are quite small for each population and unpredictable in structure. Finally, we consider the time-averaged ratio between the total excitatory and total inhibitory input into each neuron, which we will refer to as the *E/I ratio*. We plot in Figure 1C a histogram of the E/I ratios across the network, which shows relatively tight clustering about a mean near −1. Since the excitatory and inhibitory inputs are nearly equal in magnitude for each neuron in this case, the total drive is quite small in magnitude across the network and the collective dynamics are thus well balanced.

### 2.2. One-Population Model Violating Dale's Law

In contrast to the conventional balanced network modeling framework that splits the full neuronal network into excitatory and inhibitory subpopulations to obey Dale's law, we now develop a single population network model in which each neuron is able to send out excitatory signals to a subset of its post-synaptic neighbors and inhibitory signals to other post-synaptic neighbors. In this case, the state of the *i*th neuron in the single population at time *t* is governed by


(3) $$\begin{array}{l} \sigma^{i}\left(t\right)=H\left(\mu^{i}\left(t\right)-\theta \right), \end{array}$$

where the subscripts from the previous section are dropped since only one population is considered, and all terms without subscripts are given the same interpretations as described in the previous section. The total drive μ^*i*^(*t*) into the *i*th neuron at time *t* is


(4) $$\begin{array}{l} \mu^{i}\left(t\right)=\overset{N}{\underset{j=1}{\sum }}J^{ij}\sigma^{j}\left(t\right)+\mu^{0}, \end{array}$$

where *J*^*ij*^ denotes the connection strength between the *i*th post-synaptic neuron and the *j*th pre-synaptic neuron and $\mu^{0}=f_{E}m_{0}\sqrt{K}$ is the total external input into a neuron. Reflecting the potential for each neuron to provide both excitation and inhibition, the recurrent connection strength *J*^*ij*^ is now chosen to be $J_{E}/\sqrt{K}$ with probability *K*/*N*, $-J_{I}/\sqrt{K}$ with probability *K*/*N*, and 0 otherwise. As in the two-population network, each neuron is expected to receive *K* excitatory and *K* inhibitory incoming synaptic connections. Positive parameters *J*_*E*_ and *J*_*I*_ determine the relative strengths of the excitatory and inhibitory connections, respectively.

Since this one-population network model contains both positive and negative feedback loops, with inhibition and excitation of the same order of magnitude, it is intuitive to hypothesize that balanced dynamics may be achieved under appropriate constraints on the network structure. However, since both feedback types are simultaneously provided by a single computational unit in this case, whether the lack of separation in the control mechanisms impacts the robustness of the balanced operating state and its corresponding parameter regime will be investigated in the next section. Moreover, we will later examine if there are any functional differences between the one-population and two-population models, discussing potential reasons for why nature has largely selected the two-population setting that obeys Dale's law for neuronal networks in particular.

## 3. Results

### 3.1. Balanced Network Dynamics in the One-Population Model

To address the question of whether the one-population model demonstrates balanced dynamics akin to the classical two-population model, we first derive the theoretical parameter regime for which the one-population network activity is indeed balanced. To achieve asynchronous dynamics, the balanced operating state requires the long-time mean activity of the population to remain positive and less than 1 in the large-network limit. Hence, we require 0 < *m* < 1 as *N* → ∞ and as *K* → ∞, with sparse connectivity such that 1 ≪ *K* ≪ *N*. We will show that this requirement yields theoretical bounds on the model parameters necessary in order to achieve balanced dynamics. In simulations that utilize finite network realizations, these bounds hold approximately so long as the network size is sufficiently large.

We consider the population-averaged total drive into the *i*th neuron in the one-population network, $E\left[\mu^{i}\left(t\right)\right]=E\left[\left(\sum_{j=1}^{N}J^{ij}\sigma^{j}\left(t\right)\right)+\mu^{0}\right],$ and use it to determine an analytical expression for the mean activity necessary to derive balance conditions. Assuming that the synaptic connections and states are independent, since correlations are weak in the large-network limit, $E\left[\left(\sum_{j=1}^{N}J^{ij}\sigma^{j}\left(t\right)\right)\right]=\sum_{j=1}^{N}E\left[J^{ij}\right]E\left[\sigma^{j}\left(t\right)\right]$. Since each neuron is expected to receive a total of *K* incoming connections of each type and because the population-averaged state is *E*[σ^*j*^(*t*)] = *m*(*t*) regardless of neuron index, the population-averaged drive reduces to


$$\begin{array}{l} E\left[\mu^{i}\left(t\right)\right]=\overset{N}{\underset{j=1}{\sum }}\left(\frac{J_{E}}{\sqrt{K}}\frac{K}{N}-\frac{J_{I}}{\sqrt{K}}\frac{K}{N}\right)E\left[\sigma^{j}\left(t\right)\right]+\mu^{0}.\\ \text{\#x000A0; }=\sqrt{K}\left(\left(J_{E}-J_{I}\right)m\left(t\right)+f_{E}m_{0}\right). \end{array}$$

Computing the time-average in the long-time limit, such that μ_*i*_(*t*) → μ and *m*(*t*) → *m*, yields


(5) $$\begin{array}{l} \mu =\sqrt{K}\left(\left(J_{E}-J_{I}\right)m+f_{E}m_{0}\right). \end{array}$$

The long-time, time-averaged mean total input is, therefore, at most $O\left(\sqrt{K}\right)$. However, this implies that the time-averaged mean inputs potentially increase with network size and must be dynamically adjusted in order for the network to exhibit asynchronous and irregular dynamics. It is, therefore, necessary for the total excitatory and total inhibitory inputs to approximately cancel for balanced dynamics to be possible in the large-network limit. For μ to be O(1), we require


(6) $$\begin{array}{l} \left(J_{E}-J_{I}\right)m+f_{E}m_{0}=O\left(1/\sqrt{K}\right), \end{array}$$

which must vanish in the large-network limit. In this case, the resultant theoretical time-averaged mean activity is


(7) $$\begin{array}{l} m=\frac{f_{E}}{\left(J_{I}-J_{E}\right)}m_{0}, \end{array}$$

which linearly grows with the overall external input strength parameter *m*_0_. Since the mean activity needs to be positive for biological realism and *f*_*E*_ > 0, we obtain the balance condition


(8) $$\begin{array}{l} J_{I}>J_{E}. \end{array}$$

**Figure 2:**
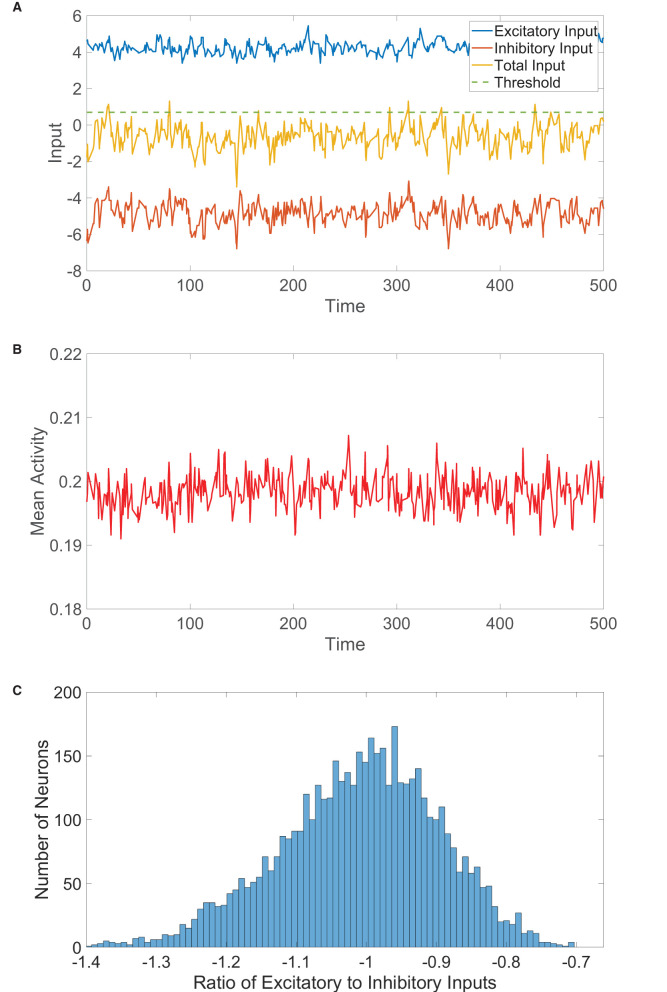
One-population balanced network dynamics. **(A)** Excitatory (blue), inhibitory (red), and net (yellow) inputs into a sample neuron in a one-population balanced network. The dashed (green) line indicates the firing threshold. **(B)** The population-averaged state (mean activity) of the single population as a function of time. **(C)** Histogram of the ratio between time-averaged excitatory and inhibitory inputs across the one-population neuronal network. In evolving the state of each neuron in the one-population network, the mean time between subsequent updates is τ = 10 ms unless stated otherwise. Parameters utilized are *J*_*E*_ = 1, *J*_*I*_ = 1.5, *N* = 5, 000, *f*_*E*_ = 0.5, *m*_0_ = 0.2, *K* = 200, and θ = 0.7.

Intuitively, since the external input is excitatory, the synaptic inputs generated from within the network must be inhibition dominated to dynamically cancel the positive external input and produce irregular dynamics. In Figure 2A, we see that when the balance condition is satisfied, large excitatory and inhibitory inputs indeed cancel over time to produce irregular dynamics and infrequent firing events for a sample neuron in the single-population model.

The variance of the synaptic inputs across the one-population network is also bounded in the large-network limit. With justification analogous to the computation of the population-averaged drive, the long-time limit of the input variance for an arbitrary *i*th neuron is


$$\begin{array}{l} var\left(\mu^{i}\right)=\overset{N}{\underset{j=1}{\sum }}var\left(J^{ij}\sigma^{j}\right)=\overset{N}{\underset{j=1}{\sum }}\left(E\left[{\left(J^{ij}\sigma^{j}\right)}^{2}\right]-{\left(E\left[J^{ij}\sigma^{j}\right]\right)}^{2}\right)\\ \;=\overset{N}{\underset{j=1}{\sum }}\left(E\left[{\left(J^{ij}\right)}^{2}\right]E\left[\sigma^{j}\right]-E{\left[J^{ij}\right]}^{2}E{\left[\sigma^{j}\right]}^{2}\right)\\ \;=\overset{N}{\underset{j=1}{\sum }}\left(\left(\frac{J_{E}^{2}}{K}\frac{K}{N}+\frac{J_{I}^{2}}{K}\frac{K}{N}\right)m-{\left(\frac{J_{E}}{\sqrt{K}}\frac{K}{N}-\frac{J_{I}}{\sqrt{K}}\frac{K}{N}\right)}^{2}m^{2}\right)\\ \;=\left(J_{E}^{2}+J_{I}^{2}\right)m-{\left(J_{E}-J_{I}\right)}^{2}\frac{K}{N}m^{2}. \end{array}$$

Therefore, in the limit of large *N* with *K* ≪ *N*, $var\left(\mu^{i}\right)\to \left(J_{E}^{2}+J_{I}^{2}\right)m$, remaining bounded and O(1). As in the two-population case, such finite input fluctuations about the near-zero expected total drive are of comparable order to the firing threshold and generate irregular firing activity. We verify this for a large one-population network realization in Figure 2B, observing that the dynamics are asynchronous with relatively small fluctuations in time about the nearly statistically stationary mean activity. The *E*/*I* input ratios across the network, plotted in Figure 2C, are centered around −1, as expected in the balanced state.

Similarly, in the case of the two-population network composed of excitatory and inhibitory neurons obeying Dale's law, it is necessary for both 0 < *m*_*E*_ < 1 and 0 < *m*_*I*_ < 1 to avoid synchronous or completely quiescent network dynamics. As shown in the seminal work on balanced networks in the case of two populations (van Vreeswijk and Sompolinsky, 1996, 1998), this yields the following theoretical expressions for the time-averaged mean activities in the balanced state


(9a) $$\begin{array}{l} m_{E}=\frac{|J_{II}|f_{E}-|J_{EI}|f_{I}}{J_{IE}|J_{EI}|-J_{EE}|J_{II}|}m_{0} \end{array}$$

(9b) $$\begin{array}{l} m_{I}=\frac{J_{IE}f_{E}-J_{EE}f_{I}}{J_{IE}|J_{EI}|-J_{EE}|J_{II}|}m_{0}, \end{array}$$

which generates a linear scaling of the mean activities in each subpopulation with *m*_0_, as obtained for the one-population network in Equation (7).

**Figure 3:**
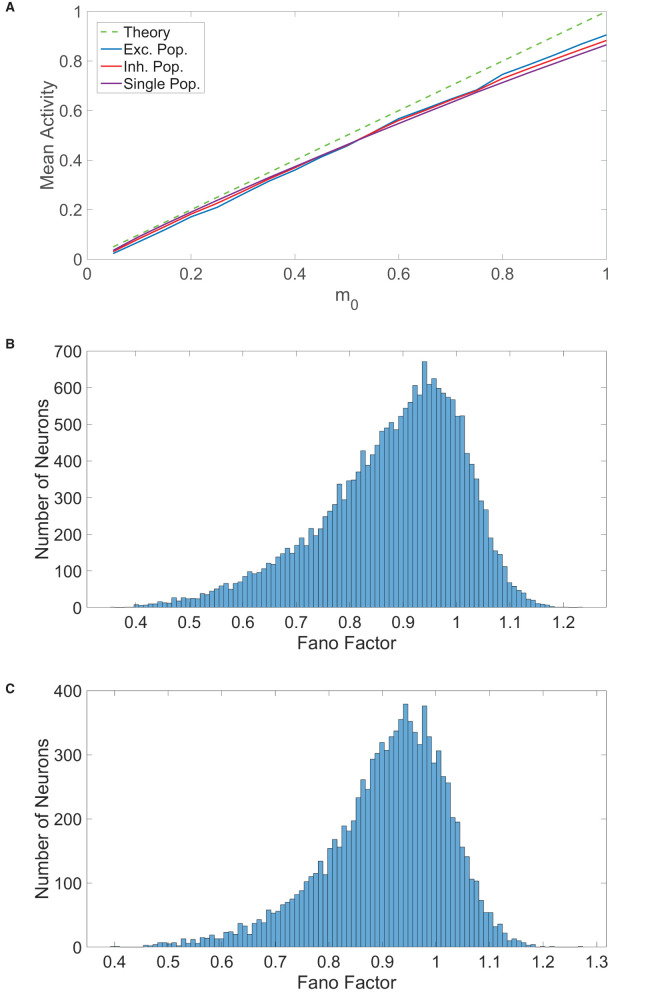
Comparison of balanced network dynamics. **(A)** The mean activity averaged over a long time horizon for various choices of external input strength *m*_0_, plotted for the excitatory (blue), inhibitory (red), and single (purple) populations. This is compared to the expected mean activity curve from balanced network theory in the large-network limit. Parameters are chosen such that the theoretical mean activity is identical for all three population types. **(B)** Histogram of the spike count Fano factors across neurons in the two-population network. **(C)** Histogram of the spike count Fano factors across neurons in the one-population network. Parameters in the one-population network utilized are *J*_*E*_ = 1, *J*_*I*_ = 1.8, *N* = 20, 000, *f*_*E*_ = 0.8, *K* = 800, and θ = 1. Parameters in the two-population network utilized are *J*_*EE*_ = *J*_*IE*_ = 1, *J*_*II*_ = −1.8, *J*_*EI*_ = −2, *N*_*E*_ = 10, 000, *N*_*I*_ = 10, 000, *f*_*E*_ = 1, *f*_*I*_ = 0.8, *m*_0_ = 0.1, *K* = 800, θ_*E*_ = 1, and θ_*I*_ = 0.8.

Requiring that both the excitatory and inhibitory time-averaged mean activities are positive and finite, the parameter bounds


(10) $$\begin{array}{l} \frac{f_{E}}{f_{I}}>\frac{\left|J_{EI}\right|}{\left|J_{II}\right|}>\frac{J_{EE}}{J_{IE}} \end{array}$$

are necessary for balanced dynamics in the large-network limit for the two-population model. In Figure 3A, we see that as the external input is increased, by adjusting *m*_0_ for different network simulations, the time-averaged mean activity for both the one-population and two-population networks demonstrates highly linear growth. These strongly linear gain curves agree well with the theoretical predictions in the large-network limit given in Equations (7) and (9). Even when Dale's law is violated, we conclude that, although the activity of the individual neurons is extremely nonlinear, the mean activity of the network exhibits linear growth with external input strength for finite yet large network realizations in the balanced state. Experimental recordings of neuronal activity often exhibit a linear increase in firing rate with external input strength (Rauch et al., 2003; La Camera et al., 2006), but here we see that adherence to Dale's law is not in fact necessary to produce such a linear response. Similarly, the irregular neuronal dynamics observed in experiment (Haider et al., 2006; Miura et al., 2007; London et al., 2010) are reproduced theoretically and in simulation for the one-population model, capturing the hallmark dynamical features of classical two-population balanced networks.

Large spike count variability across controlled trials is commonly observed in cortical neurons in evoked conditions (Shadlen and Newsome, 1994; Churchland et al., 2011), and such stochasticity in spike emission is seen in balanced networks that obey Dale's law (Tsodyks et al., 1999; Litwin-Kumar and Doiron, 2012). To compare the variability in activity for the one-population and two-population networks in the balanced state, we determine the distribution of spike count Fano factors across each network. The spike count Fano factor for a neuron over time interval [*t, t* + Δ*t*] is


$$\begin{array}{l} F\left(t,t+\Delta t\right)=\frac{var\left(N\left(t,t+\Delta t\right)\right)}{E\left[N\left(t,t+\Delta t\right)\right]}, \end{array}$$

which gives the ratio of the variance to the mean for the number of times the neuron fires over this time window, *N*(*t, t* + Δ*t*), across trials. In each trial for a given model, an identical network is generated with different initial conditions and each neuron in the network is updated at identical times across trials. The time window in this case is Δ*t* = 100 ms and Fano factors were computed using 100 trials. For a given neuron, we averaged the Fano factors corresponding to each time interval in a full simulation of 10000 ms to obtain a single neuronal Fano factor. We plot in Figure 3B the histogram of spike count Fano factors across neurons in the two-population network and similarly plot the Fano factor histogram for the one-population network in Figure 3C. For both network types, we observe that the mean spike count Fano factor is near 1, which is the Fano factor for a homogeneous Poisson process. Hence, we conclude that a high degree of spike count variability is indeed achievable by the one-population model violating Dale's law, facilitating rich computational capabilities similar to those of the two-population network.

### 3.2. Scaling With Network Size

Since the key dynamical features explored thus far are comparable for the one-population and two-population networks, it still remains unclear why Dale's law is typically followed in the brain if not to help maintain balance. We now investigate several additional dynamical and functional properties for which the two network models potentially differ qualitatively to shed additional light on the ubiquity of Dale's law in the brain.

**Figure 4:**
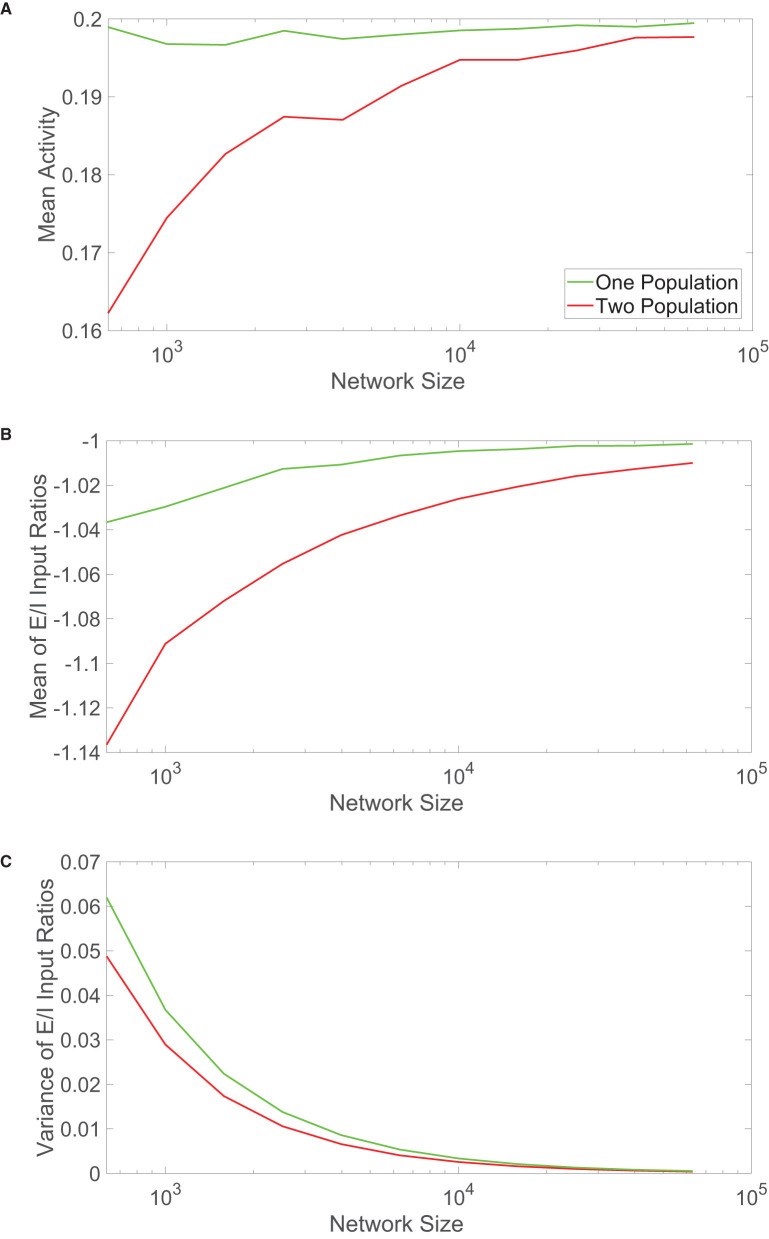
Comparison of scaling properties with network size. **(A)** The time-averaged mean activity as a function of the total network size for the one-population (green) and two-population (red) networks. The mean activity for each population expected from theory is 0.2. **(B)** Mean of the ratio between time-averaged excitatory and inhibitory inputs across each network model as a function of network size. **(C)** Variance of the ratio between time-averaged excitatory and inhibitory inputs across each network model as a function of network size. Parameters in the one-population network utilized are *J*_*E*_ = 1, *J*_*I*_ = 1.5, *m*_0_ = 0.2, *f*_*E*_ = 0.5, *K* = 0.08*N*, and θ = 0.7. Parameters in the two-population network utilized are *J*_*EE*_ = *J*_*IE*_ = 1, *J*_*II*_ = −1.5, *J*_*EI*_ = −5/3, *f*_*E*_ = 1, *f*_*I*_ = 0.8, *m*_0_ = 0.2, *K* = 0.08*N, N*_*E*_ = *N*_*I*_ = 0.5*N*, θ_*E*_ = 1, and θ_*I*_ = 0.8. Each plot is averaged over 10 network realizations.

Previous sections only examined the activity of the networks theoretically in the large-network limit and compared their dynamics for relatively large-scale network realizations. Considering other biological systems that disobey Dale's law are often significantly smaller than brain networks, such as colonies of honeybees charged with similar functional tasks as neuronal networks, we compare the scaling properties of the two network models as the number of constituent neurons is increased. In Figure 4A, we plot the time-averaged mean activity across each network as a function of the network size. We observe that for relatively small networks, with less than ten thousand neurons, the mean activity for the two-population model remains below the theoretical prediction and grows with network size. On the other hand, the mean activity of the one-population model remains nearly constant and close to the value expected theoretically over the same range of network sizes.

We analogously plot the mean and variance of the E/I input ratios with growing network sizes in Figures 4B,C, respectively. Particularly for small network sizes, the one-population network exhibits mean E/I input ratios closer to −1 than seen for the two-population model. The variances are relatively comparable across network sizes, with the one-population network displaying slightly more variability in the E/I input ratios across the network.

These scaling properties together suggest that the one-population model better maintains balanced dynamics for smaller network sizes, with comparable performance once the networks are sufficiently large. Considering that the results in Figure 4 are averaged over 10 different network realizations for each network size, this trend is robust and largely independent of a specific network simulation. For smaller networks outside of the realm of neuroscience, it is possible that evolution has selected systems that violate Dale's law in order to better achieve balanced dynamics and the accompanying functional benefits of the balanced operating state. While a computational unit in the one-population network has more diversity in functionality and may require a larger energetic cost, if the network is of moderate size, the price is likely worthwhile to pay for maintaining balanced dynamics. This leaves the remaining question of why neuronal networks primarily obey Dale's law if in the large-network limit, which well applies for the brains of complex species, the two network types thus far exhibit similar dynamical features. We explore this question in the context of two common functional tasks in the subsequent sections.

### 3.3. Fast Tracking of Inputs

A major functional advantage of the balanced state in the two-population setting is the ability to rapidly respond to changes in external input (van Vreeswijk and Sompolinsky, 1998). In particular, an $O\left(1/\sqrt{K}\right)$ change in external input scaling strength *m*_0_ results in a comparable adjustment in the steady-state mean activity for each population in accordance with Equation (9). We determine how the fast-tracking ability of the one-population model compares to that of the classical two-population network, underlining a qualitative functional difference between the two networks.

**Figure 5:**
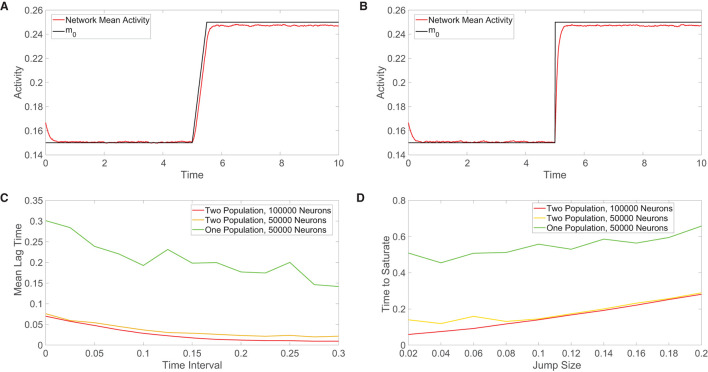
Comparison of the tracking of network inputs. **(A)** Mean activity in the one-population model plotted over time (red) for an external input that evolves continuously in time. The external input strength parameter *m*_0_ varies over time and is plotted for comparison (black), while all other parameters are fixed. Here the external input is either constant or linearly increases in time. **(B)** Analogous plot as in **(A)**, except now the external input changes discontinuously at *t* = 5 *via* a jump discontinuity and remains constant otherwise. The same range of *m*_0_ values is used in each plot. **(C)** Across a family of linearly-ramped inputs of the form in panel **(A)**, the time interval over which the external input transitions from its fixed minimal value to its fixed maximal value is varied. For each such time interval, the mean lag time between the network response and the external input is plotted. The mean lag time plotted for each input is the time difference between the mean activity and the external input averaged from when the external input first begins to change in time until the network mean activity saturates (reaches the value of the long-time, time-averaged mean activity following input saturation). **(D)** Across a family of discontinuous inputs of the form in **(B)**, the maximal value of the external input is varied. For each resultant input jump size, the time elapsed from the input jump until the network mean activity saturates is plotted. In **(C,D)**, the fast-tracking properties are plotted in red for the two-population network with 100, 000 neurons, in orange for the two-population network with 50, 000 neurons, and in green for the one-population network with 50000 neurons. Parameters in the one-population network utilized are *J*_*E*_ = 1, *J*_*I*_ = 1.5, *m*_0_ = 0.2, *f*_*E*_ = 0.5, *K* = 0.08*N*, and θ = 0.7. Parameters in the two-population network utilized are *J*_*EE*_ = *J*_*IE*_ = 1, *J*_*II*_ = −1.5, *J*_*EI*_ = −5/3, *f*_*E*_ = 1, *f*_*I*_ = 0.8, *m*_0_ = 0.2, *K* = 0.08*N, N*_*E*_ = *N*_*I*_ = 0.5*N*, θ_*E*_ = 1, and θ_*I*_ = 0.8. The mean activity is computed over 0.01 time windows in each case and the plots in **(C,D)** are averaged over 10 network realizations.

We consider the one-population network response to two classes of time-varying external inputs, and then compare the response speeds for the different network models across a family of external inputs of each class. In Figure 5A, the external input continuously increases in a linear fashion within a small time window and otherwise remains constant. For even a relatively high input slope, we see that the one-population network activity nearly identically follows the external input over all times, as expected from balanced network theory. We similarly address the case in which the external input instead demonstrates a jump discontinuity in Figure 5B, which can be viewed as the high-slope limit of the previous linearly-ramped input. The mean activity of the one-population network demonstrates a minor deviation from the external input immediately following the jump, but it calibrates to the new magnitude of the input after only a short amount of time.

For the class of linearly-ramped inputs akin to Figure 5A, we investigate how the response of each network model depends on the time interval over which the input linearly increases to achieve its fixed maximal value. We note that as this time interval becomes longer, the input increases more slowly. We compute the mean lag time across a family of such linearly-ramped inputs. To measure the lag time in the tracking of each external input, we compute the difference between the time when each external input value is realized and the time when the corresponding value of the network mean activity is reached, averaging over this time difference from when the external input first begins to change until the network activity saturates in order to obtain the mean lag time. For both the one-population and two-population networks, we observe in Figure 5C that the mean lag time decreases with the size of the time interval over which the input is linearly ramped, demonstrating less lag for inputs that change more slowly as expected. However, for each external input, the two-population network exhibits less lag than the one-population network, especially for more rapidly varying inputs.

For the discontinuous class of inputs represented in Figure 5B, we similarly investigate how the external input tracking ability of each network is affected by the size of the jump in *m*_0_, which determines the resultant magnitude of the instantaneous input change that the network must respond to and also the maximal external input value. Since the input jumps instantaneously, the prior method for measuring the time difference between the network response and input must be modified. In this case, for each external input, we compute the amount of time following the jump that elapses until the network mean activity saturates; here shorter saturation time is evidence of a faster response. In Figure 5D, we see that as the jump size increases, the two network models require more time to saturate in response to the new external input value. As observed for the continuous class of external inputs, the two-population network demonstrates superior tracking ability with a shorter saturation time for each discontinuous input utilized.

This trend is true when the different network models each have the same total number of neurons as well as when the excitatory and inhibitory populations in the two-population model each have the same total number of neurons as the entire one-population network. Based on the diversity in the types of outputs that neurons can transmit to their neighbors, it can be argued that a neuron in the two-population network has less computational capability than a neuron in the one-population network. For this reason, we consider such a comparison in tracking performance when the two-population network has a larger total number of neurons. While doubling the number of neurons in the two-population network improves external input tracking, the gains depicted in Figure 5 are relatively marginal. We remark that in comparing the scaling properties for the one-population networks with two-population networks containing double the neurons in Figure 4, the overall trend of more balanced dynamics in the one-population model for small network sizes and comparable performance in the large-network limit still holds. Hence, the potentially high energetic costs of the large synaptic input currents necessary to maintain balanced dynamics garner a rapid response to changes in external inputs for both network types, but we hypothesize that evolution may have selected two-population networks obeying Dale's law in the brain in order to facilitate yet further improved input tracking necessary to respond to stimuli and make decisions over very short time scales. For smaller networks outside of the brain, where the response need not be quite so fast yet still efficient enough to carry out network functions, the one-population architecture likely suffices.

### 3.4. Decision-Making Dynamics

Activity in multiple brain areas, including the prefrontal cortex, thalamus, basal ganglia, and parietal cortex, plays an important role in decision-making (Platt and Glimcher, 1999; Munakata et al., 2011; Ding and Gold, 2013), and numerous studies have made significant parallels between the collective decision-making mechanisms used by social insects and neurons in the brain (Couzin, 2009; Marshall et al., 2009; Seeley et al., 2012). For each biological system, when a certain cluster of units displays sufficiently high activity relative to other competing groups, a decision is typically made. The classical leaky competing accumulator model for decision-making facilitates the competition between neuronal assemblies by directly including lateral inhibition between clusters (Usher and McClelland, 2001), and an attractor such that one cluster demonstrates significantly more activity than the others corresponds to a particular decision. Balanced networks containing excitatory and inhibitory neurons that incorporate competing pools of neurons are known to demonstrate such decision-making dynamics (Wang, 2008; Cohen et al., 2019), and we conclude by investigating if one-population networks violating Dale's law have analogous functionality.

We consider two competing pools of one-population neurons, *A* and *B*, with dynamics and connectivity within each pool as described in Section 2.2. We relabel the recurrent connection strengths within the *k*th pool as $J_{E}^{kk}$ and $J_{I}^{kk}$ (here *k* = *A, B*), to distinguish them from the cross-pool connections from the *l*th pool to the *k*th pool, whose strengths are similarly denoted $J_{E}^{kl}$ and $J_{I}^{kl}$. We assume that each pool contains the same number of neurons, *N*, and that the cross-pool connection strength $J_{kl}^{ij}$ between the *i*th post-synaptic neuron in the *k*th pool and the *j*th pre-synaptic neuron in the *l*th pool is chosen to be $J_{E}^{kl}/\sqrt{K}$ with probability *K*/*N*, $-J_{I}^{kl}/\sqrt{K}$ with probability *K*/*N*, and 0 otherwise. As a result, each neuron is expected to receive *K* excitatory and *K* inhibitory incoming synaptic connections from within its own pool and is also expected to receive the same number of incoming cross-pool connections of each type.

In facilitating an attractor state where relatively high activity in one pool corresponds to a particular decision, we assume that the external input into the *k*th pool is now scaled by parameter *f*_*k*_ rather than *f*_*E*_ as considered earlier, with the overall external input scaling strength *m*_0_ identical for each pool. For successful decision-making dynamics, we require that when *f*_*A*_ > *f*_*B*_, the mean activity of pool *A* is greater than the mean activity of pool *B*, namely *m*_*A*_ > *m*_*B*_.

Following an argument similar to Section 3.1, the long-time, population-averaged total drive into an arbitrary neuron in pool *A* and in pool *B* of the one-population network, respectively, is


$$\begin{array}{r} E\left[\mu_{A}\right]=\sqrt{K}\left(\left(J_{E}^{AA}-J_{I}^{AA}\right)m_{A}+\left(J_{E}^{AB}-J_{I}^{AB}\right)m_{B}+m_{0}f_{A}\right)\\ \equiv \sqrt{K}\left(J_{in}^{AA}m_{A}+J_{out}^{AB}m_{B}+m_{0}f_{A}\right).\\ E\left[\mu_{B}\right]=\sqrt{K}\left(J_{in}^{BB}m_{B}+J_{out}^{BA}m_{A}+m_{0}f_{B}\right) \end{array}$$

In order for the dynamics to be balanced theoretically, it is necessary for *E*[μ_*A*_] and *E*[μ_*B*_] to remain O(1) in the large-network limit, requiring that


(11a) $$\begin{array}{l} J_{in}^{AA}m_{A}+J_{out}^{AB}m_{B}+m_{0}f_{A}=0 \end{array}$$

(11b) $$\begin{array}{l} J_{in}^{BB}m_{B}+J_{out}^{BA}m_{A}+m_{0}f_{B}=0 \end{array}$$

We assume symmetry in the connectivity parameters for the two pools, such that $J_{in}^{AA}=J_{in}^{BB}\equiv J_{in}$ and $J_{out}^{AB}=J_{out}^{BA}\equiv J_{out}$. This implies that the main determining factor in the decision-making process is the difference in the external input scalings for the pools, *f*_*A*_ and *f*_*B*_.

Solving linear system (11) yields the time-averaged mean activity for each pool


(12a) $$\begin{array}{l} m_{A}=\frac{J_{in}f_{A}-J_{out}f_{B}}{J_{out}^{2}-J_{in}^{2}}m_{0} \end{array}$$

(12b) $$\begin{array}{l} m_{B}=\frac{J_{in}f_{B}-J_{out}f_{A}}{J_{out}^{2}-J_{in}^{2}}m_{0}. \end{array}$$

For balanced dynamics among at least one of the competing pools, it is required that 0 < *m*_*A*_ < 1 or 0 < *m*_*B*_ < 1. We assume that in the absence of any communications between the pools, each is internally balanced. From the analysis in Section 3.1, we conclude that $J_{I}^{kk}>J_{E}^{kk}$ and, therefore, *J*_*in*_ < 0. The cross-pool connection strength is the remaining parameter that determines whether balanced dynamics are achieved and whether the network carries out successful decision-making as well. We argue that this is determined by the sign of *J*_*out*_ and how the magnitude of *J*_*out*_ compares to |*J*_*in*_|.

For the mean activity of pool *A* to increase with *f*_*A*_ and the mean activity of pool *B* to increase with *f*_*B*_, as expected in effective decision-making, it is necessary for |*J*_*in*_| > |*J*_*out*_|. Similarly, for increased input into the competing pool *B* to suppress the mean activity of pool *A*, it is also necessary for *J*_*out*_ < 0. When *f*_*A*_ > *f*_*B*_, this means that pool *A* is dominant with balanced dynamics such that 0 < *m*_*A*_ < 1, whereas pool *B* is either relatively suppressed or quiescent. Winner-take-all dynamics emerge when unequal alternatives are presented, and the pool with greater external input is capable of exhibiting nonzero mean activity with balanced dynamics while the other pool is fully suppressed. Collectively, this yields the following conditions for effective decision-making in competing pools of single-population neurons


(13a) $$\begin{array}{l} J_{out}<0,J_{in}<0 \end{array}$$

(13b) $$\begin{array}{l} |J_{in}|>|J_{out}|. \end{array}$$

**Figure 6:**
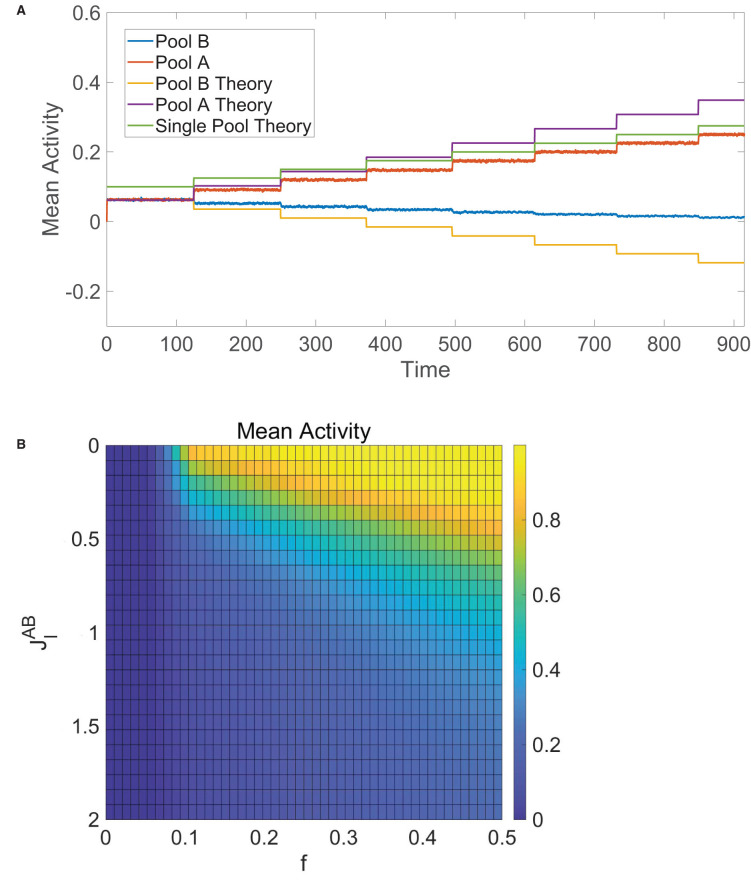
Decision-making in competing pools of one-population neurons. **(A)** Mean activity for pool *B* (blue) and pool *A* (red) over time, where the input into pool *B* is fixed with *f*_*B*_ = 0.1 and the input into pool *A* has *f*_*A*_ = 0.1 initially with *f*_*A*_ stepped up by 0.025 at several times. These simulated mean activities are compared to the theoretical mean activities in Equation (12) for pool *B* (orange) and pool *A* (purple) as well as the theoretical mean activity for a single pool of one-population neurons (green) given by Equation (7). **(B)** Time-averaged mean activity for pool *A* in the tied alternatives case as a function of external input scaling strength *f*_*A*_ = *f*_*B*_ = *f* and cross-pool inhibition strength $J_{I}^{AB}=J_{I}^{BA}$, varied across runs. Unless specified as varying, the parameters utilized are *N* = 10, 000 neurons in each pool, $J_{E}^{AA}=J_{E}^{BB}=1,J_{I}^{AA}=J_{I}^{BB}=1.8,J_{E}^{AB}=J_{E}^{BA}=1,J_{I}^{AB}=J_{I}^{BA}=1.5,k=400,f_{B}=0.1,$ and θ = 1.

We verify this numerically in Figure 6A, where initially *f*_*A*_ = *f*_*B*_ and then *f*_*A*_ jumps in value at several points in time. We see that the mean activities are equal and well agree with Equation (12) at the start of the simulation. As *f*_*A*_ is first increased, the mean activity of pool *A* increases whereas the mean activity of pool *B* decreases yet remains nonzero, agreeing with theoretical predictions from Equation (12) as well. Once *f*_*A*_ is sufficiently large, pool *B* becomes completely quiescent and dynamics of pool *A* are akin to the single-population balanced network theory for one pool given by Equation (7), since pool *A* is now effectively receiving no input from pool *B*.

In the special case of tied alternatives, when *f*_*A*_ = *f*_*B*_ = *f*, we note that Equation (12) reduces to $m_{A}=m_{B}=\frac{1}{-J_{out}-J_{in}}fm_{0}$. Under the same parameter restrictions in Equation (13), the mean activity of each pool in this case increases with external stimulus scaling *fm*_0_ and decreases with the magnitude of the effective internal recurrent connectivity strength, |*J*_*in*_|, and the magnitude of the effective cross-cluster connectivity strength, |*J*_*out*_|. These trends can be verified numerically in Figure 6B, which tracks the time-averaged mean activity for pool *A* in the case of tied alternatives across simulations where the external input scaling, *f*, and cross-pool inhibition strength, $J_{I}^{AB}=J_{I}^{BA}$, are varied. Theoretically, balanced dynamics can persist as long as −*J*_*out*_ − *J*_*in*_ > *fm*_0_, particularly when there is enough total recurrent inhibition between the neurons to counteract the excitatory external stimulus.

When the balance conditions in Equation (13) are satisfied, the one-population network with competing pools generally functions as follows: (1) under spontaneous conditions when *f*_*A*_ = *f*_*B*_, both pools demonstrate balanced dynamics that are responsive to changes in external input and (2) under evoked conditions when *f*_*A*_ ≠ *f*_*B*_, the pool with a stronger external stimulus, signaling a more profitable alternative, continues to demonstrate balanced dynamics while the other pool is suppressed. Considering systems violating Dale's law are often required to effectively make decisions, though perhaps not as rapidly as neuronal networks in the brain, this functionality agrees with real-world constraints.

## 4. Discussion

While theoretical studies largely assume strict adherence to Dale's law, in light of notable exceptions in the brain and other biological systems with complex functionality, we have systematically studied the computational capabilities of neuronal networks that instead violate Dale's law. We demonstrated that, like classical two-population networks composed of excitatory and inhibitory subpopulations, one-population networks with neurons that can transmit both excitatory and inhibitory signals are capable of exhibiting balanced network dynamics resembling the irregular and asynchronous activity commonly observed in experiment. These one-population networks rapidly respond to changes in inputs and mediate effective decision-making dynamics in the balanced regime. In the case of smaller systems, the one-population networks in fact exhibit a higher degree of balance than two-population networks of the same size, suggesting a potential reason why systems with less computational units than the brain, such as honeybee colonies, do not adhere to Dale's law even though they operate under similar computational goals. However, for larger systems, where the dynamics are sufficiently balanced for both network models, the two-population networks demonstrate superior fast-tracking of inputs and this suggests that evolution may have constructed neuronal systems that primarily obey Dale's law in order to prioritize the particularly rapid information processing necessary for survival.

This work aimed to examine the fundamental mechanistic properties imbued by the presence or absence of a Dale's law constraint, abstracting over more detailed biological assumptions to isolate the impact of this specific network characteristic. Focusing on network computations imparted by firing patterns as opposed to subthreshold voltage dynamics, each individual neuron in our analysis possessed only two possible states. However, there is a rich history of studies demonstrating analogous balanced dynamics for networks with more detailed single-neuron models (Renart et al., 2007; Rosenbaum and Doiron, 2014), and we expect nearly identical comparisons to be drawn for neurons with integrate-and-fire dynamics. Prior work shows that in the classical two-population setting, both binary-state and integrate-and-fire balanced neuronal networks furnish the same input-output mapping structure (Barranca et al., 2019b; Gu et al., 2019). Since the presence of other systems capable of complex computations, such as honeybee colonies and artificial neural networks, was a motivation for this work, an investigation of Dale's law in the context of single-unit dynamics more faithful to such systems, as opposed to the brain, would be a natural avenue for further study. While numerous recent advances in neuromorphic computing demonstrate biologically plausible mechanisms for learning and transmitting error information (Lillicrap et al., 2016; Barranca, 2021; Yang et al., 2021), this work does not directly seek to improve upon state-of-the-art methods. Instead, this study shows that, from a functional perspective, strict adherence to Dale's law does not necessarily significantly diminish performance and rather suggests that for artificial neural networks with a particularly large number of units some efficiencies may be gleaned from connectivity structures inspired by two-population balanced networks.

There is a host of different excitatory and inhibitory neurotransmitters in the brain (Yamada et al., 1989; McCormick et al., 1993; Koch, 1999), and though we have neglected their individual time-scales and the relative magnitude of their effects, it is possible to similarly construct network models violating Dale's law that are capable of sending out distinct sets of neurotransmitters to different neighbors. The release of a diversity of neurotransmitters by a single neuron likely grants flexibility to circuits in the brain, but the maintenance of a mechanism for selective neurotransmitter release may require a higher metabolic cost. It would be especially informative to explore an energy landscape, where the addition of neurons obeying or violating Dale's law incur different energetic costs, and determine whether the relatively rare addition of neurons violating Dale's law as found in the brain indeed optimizes certain aspects of the network performance.

While we had assumed statistically homogeneous random connectivity to be faithful to traditional balanced network theory and to facilitate analytical tractability, neuronal networks observed in experiment often exhibit complex network structure that can intimately impact the network dynamics (Massimini et al., 2005; Bonifazi et al., 2009; Markov et al., 2013). An indirect inhibitory circuit, mediated by an intermediate inhibitory neuron post-connected to an excitatory neuron, for example, may effectively imbue function like a one-population neuron capable of sending out both excitatory and inhibitory signals in certain computations (Shpiro et al., 2007). Nevertheless, how this impacts more subtle functional properties affected by time lags and precise network structure in comparison to an analogous one-population model remains unexplored. It is also possible to similarly engineer one-population networks with the goal of effectively choosing among a large number of alternatives (Ganguli et al., 2008; Heekeren et al., 2008; Barranca et al., 2019a), and we expect such networks exhibit similar functional properties as we have demonstrated in the case of two options. Our analysis focused primarily on stimulus response properties and decision making, though it would be informative to study the computational properties of one-population networks in the context of other roles, such as predictive coding and short-term memory (Whalley, 2013; Ingrosso and Abbott, 2019). Since neurotransmitters and their co-transmission have been implicated in brain disorders, including Parkinson's disease (Svensson et al., 2018), the continued study of the functional consequences of neuroatypical characteristics, such as an imbalance of excitatory and inhibitory inputs or a surplus of neurons violating Dale's law, may chart out important advances in treating neurological disorders.

## Data Availability Statement

The original contributions presented in the study are included in the article/supplementary material, further inquiries can be directed to the corresponding author.

## Author Contributions

All authors listed have made a substantial, direct, and intellectual contribution to the work and approved it for publication.

## Funding

This work was supported by NSF DMS-1812478 (VB) and by a Swarthmore Faculty Research Support Grant (VB).

## Conflict of Interest

The authors declare that the research was conducted in the absence of any commercial or financial relationships that could be construed as a potential conflict of interest.

## Publisher's Note

All claims expressed in this article are solely those of the authors and do not necessarily represent those of their affiliated organizations, or those of the publisher, the editors and the reviewers. Any product that may be evaluated in this article, or claim that may be made by its manufacturer, is not guaranteed or endorsed by the publisher.

## References

[B1] Arenas A. Diaz-Guilera A. Perez-Vicente C. J. (2006). Synchronization reveals topological scales in complex networks. Phys. Rev. Lett. 96, 114102. 10.1103/PhysRevLett.96.114102 16605825

[B2] Barranca V. J. (2021). Neural network learning of improved compressive sensing sampling and receptive field structure. Neurocomputing 455, 368–378. 10.1016/j.neucom.2021.05.061

[B3] Barranca V. J. Huang H. Kawakita G. (2019a). Network structure and input integration in competing firing rate models for decision-making. J. Comput. Neurosci. 46, 145–168. 10.1007/s10827-018-0708-6 30661144

[B4] Barranca V. J. Huang H. Li S. (2019b). The impact of spike-frequency adaptation on balanced network dynamics. Cogn. Neurodyn. 13, 105–120. 10.1007/s11571-018-9504-2 30728874 PMC6339860

[B5] Barranca V. J. Zhou D. (2019). Compressive sensing inference of neuronal network connectivity in balanced neuronal dynamics. Front. Neurosci. 13, 1101. 10.3389/fnins.2019.01101 31680835 PMC6811502

[B6] Barranca V. J. Zhou D. Cai D. (2015). Low-rank network decomposition reveals structural characteristics of small-world networks. Phys. Rev. E 92, 062822. 10.1103/PhysRevE.92.062822 26764759

[B7] Bassett D. S. Greenfield D. L. Meyer-Lindenberg A. Weinberger D. R. Moore S. W. Bullmore E. T. (2010). Efficient physical embedding of topologically complex information processing networks in brains and computer circuits. PLoS Comput. Biol. 6, e1000748. 10.1371/journal.pcbi.1000748 20421990 PMC2858671

[B8] Boccaletti S. Latora V. Moreno Y. Chavez M. Hwang D.-U. (2006). Complex networks: Structure and dynamics. Phys. Rep. 424, 175–308. 10.1016/j.physrep.2005.10.009

[B9] Boerlin M. Machens C. K. Deneve S. (2013). Predictive coding of dynamical variables in balanced spiking networks. PLoS Comput. Biol. 9, e1003258. 10.1371/journal.pcbi.1003258 24244113 PMC3828152

[B10] Bonifazi P. Goldin M. Picardo M. A. Jorquera I. Cattani A. Bianconi G.. (2009). GABAergic hub neurons orchestrate synchrony in developing hippocampal networks. Science 326, 1419–1424. 10.1126/science.1175509 19965761

[B11] Borofsky T. Barranca V. J. Zhou R. von Trentini D. Broadrup R. L. Mayack C. (2020). Hive minded: like neurons, honey bees collectively integrate negative feedback to regulate decisions. Anim. Behav., 168:33–44. 10.1016/j.anbehav.2020.07.023

[B12] Chan T.-H. Jia K. Gao S. Lu J. Zeng Z. Ma Y. (2015). Pcanet: a simple deep learning baseline for image classification? IEEE Trans. Image Process. 24, 5017–5032. 10.1109/TIP.2015.2475625 26340772

[B13] Churchland A. K. Kiani R. Chaudhuri R. Wang X. J. Pouget A. Shadlen M. N. (2011). Variance as a signature of neural computations during decision making. Neuron 69, 818–831. 10.1016/j.neuron.2010.12.037 21338889 PMC3066020

[B14] Cohen B. P. Chow C. C. Vattikuti S. (2019). Dynamical modeling of multi-scale variability in neuronal competition. Commun. Biol. 2, 319. 10.1038/s42003-019-0555-7 31453383 PMC6707190

[B15] Couzin I. D. (2009). Collective cognition in animal groups. Trends Cogn. Sci. 13, 36–43. 10.1016/j.tics.2008.10.002 19058992

[B16] Deng L. Yu D.. (2014). Deep learning: methods and applications. Found. Trends Signal Process. 7, 197–387. 10.1561/2000000039

[B17] Ding L. Gold J. I. (2013). The basal ganglia's contributions to perceptual decision making. Neuron 79, 640–649. 10.1016/j.neuron.2013.07.042 23972593 PMC3771079

[B18] Estrada E. Higham D. J. (2010). Network properties revealed through matrix functions. SIAM Rev. 52, 696–714. 10.1137/090761070

[B19] Faisal A. A. Selen L. P. Wolpert D. M. (2008). Noise in the nervous system. Nat. Rev. Neurosci. 9, 292–303. 10.1038/nrn2258 18319728 PMC2631351

[B20] Ganguli S. Bisley J. W. Roitman J. D. Shadlen M. N. Goldberg M. E. Miller K. D. (2008). One-dimensional dynamics of attention and decision making in LIP. Neuron 58, 15–25. 10.1016/j.neuron.2008.01.038 18400159 PMC7204626

[B21] Gomez-Rodriguez M. Leskovec J. Krause A. (2012). Inferring networks of diffusion and influence. Trans. Knowl. Disc. Data 5, 21. 10.1145/2086737.208674125825672

[B22] Gu Q. Li S. Dai W. Zhou D. Cai D. (2019). Balanced active core in heterogeneous neuronal networks. Front. Comput. Neurosci. 12, 109. 10.3389/fncom.2018.00109 30745868 PMC6360995

[B23] Hafting T. Fyhn M. Molden S. Moser M. B. Moser E. I. (2005). Microstructure of a spatial map in the entorhinal cortex. Nature 436, 801–806. 10.1038/nature03721 15965463

[B24] Haider B. Duque A. Hasenstaub A. R. McCormick D. A. (2006). Neocortical network activity in vivo is generated through a dynamic balance of excitation and inhibition. J. Neurosci. 26, 4535–4545. 10.1523/JNEUROSCI.5297-05.2006 16641233 PMC6674060

[B25] Heekeren H. R. Marrett S. Ungerleider L. G. (2008). The neural systems that mediate human perceptual decision making. Nat. Rev. Neurosci. 9, 467–479. 10.1038/nrn2374 18464792

[B26] Hubel D. Wiesel T. (1972). Laminar and columnar distribution of geniculo cortical fibers in the macaque monkey. J. Comp. Neurol. 146, 421–450. 4117368 10.1002/cne.901460402

[B27] Ingrosso A. Abbott L. F. (2019). Training dynamically balanced excitatory-inhibitory networks. PLoS ONE 14, e0220547. 10.1371/journal.pone.0220547 31393909 PMC6687153

[B28] Jonas P. Bischofberger J. Sandkühler J. (1998). Corelease of two fast neurotransmitters at a central synapse. Science 281, 419–424. 9665886 10.1126/science.281.5375.419

[B29] Kandel E. R. (1968). Dale's principle and the functional specificity of neurons, in Psychopharmacology; A Review of Progress, 1957–1967 (Washington, DC: US Government Printing Office), 385–398.

[B30] Koch C. (1999). Biophysics of Computation Oxford: Oxford University Press.

[B31] La Camera G. Rauch A. Thurbon D. Luscher H. R. Senn W. Fusi S. (2006). Multiple time scales of temporal response in pyramidal and fast spiking cortical neurons. J. Neurophysiol. 96, 3448–3464. 10.1152/jn.00453.2006 16807345

[B32] Lian Y. Grayden D. B. Kameneva T. Meffin H. Burkitt A. N. (2019). Toward a biologically plausible model of lgn-v1 pathways based on efficient coding. Front. Neural Circ. 13, 13. 10.3389/fncir.2019.00013 30930752 PMC6427952

[B33] Lillicrap T. P. Cownden D. Tweed D. B. Akerman C. J. (2016). Random synaptic feedback weights support error backpropagation for deep learning. Nat. Commun. 7, 13276. 10.1038/ncomms13276 27824044 PMC5105169

[B34] Lim S. Goldman M. S. (2014). Balanced cortical microcircuitry for spatial working memory based on corrective feedback control. J. Neurosci. 34, 6790–6806. 10.1523/JNEUROSCI.4602-13.2014 24828633 PMC4019795

[B35] Litwin-Kumar A. Doiron B. (2012). Slow dynamics and high variability in balanced cortical networks with clustered connections. Nat. Neurosci. 15, 1498–1505. 10.1038/nn.3220 23001062 PMC4106684

[B36] London M. Roth A. Beeren L. Hausser M. Latham P. E. (2010). Sensitivity to perturbations in vivo implies high noise and suggests rate coding in cortex. Nature 466, 123–127. 10.1038/nature09086 20596024 PMC2898896

[B37] Ludwig M. Leng G. (2006). Dendritic peptide release and peptide-dependent behaviours. Nat. Rev. Neurosci. 7, 126–136. 10.1038/nrn1845 16429122

[B38] Markov N. T. Ercsey-Ravasz M. Van Essen D. C. Knoblauch K. Toroczkai Z. Kennedy H. (2013). Cortical high-density counterstream architectures. Science 342, 1238406. 10.1126/science.1238406 24179228 PMC3905047

[B39] Marshall J. A. Bogacz R. Dornhaus A. Planqué R. Kovacs T. Franks N. R. (2009). On optimal decision-making in brains and social insect colonies. J. R. Soc. Interface 6, 1065–1074. 10.1098/rsif.2008.0511 19324679 PMC2827444

[B40] Massimini M. Ferrarelli F. Huber R. Esser S. K. Singh H. Tononi G. (2005). Breakdown of cortical effective connectivity during sleep. Science 309, 2228–2232. 10.1126/science.1117256 16195466

[B41] McCormick D. Wang Z. Huguenard J. (1993). Neurotransmitter control of neocortical neuronal activity and excitability. J. Neurophysiol. 68, 387–398. 7903176 10.1093/cercor/3.5.387

[B42] McCormick D. A. Connors B. W. Lighthall J. W. Prince D. A. (1985). Comparative electrophysiology of pyramidal and sparsely spiny stellate neurons of the neocortex. J. Neurophysiol. 54, 782–806. 2999347 10.1152/jn.1985.54.4.782

[B43] Miura K. Tsubo Y. Okada M. Fukai T. (2007). Balanced excitatory and inhibitory inputs to cortical neurons decouple firing irregularity from rate modulations. J. Neurosci. 27, 13802–13812. 10.1523/JNEUROSCI.2452-07.2007 18077692 PMC6673628

[B44] Munakata Y. Herd S. A. Chatham C. H. Depue B. E. Banich M. T. O'Reilly R. C. (2011). A unified framework for inhibitory control. Trends Cogn. Sci. (Regul. Ed.) 15, 453–459. 10.1016/j.tics.2011.07.011 21889391 PMC3189388

[B45] Newman M. E. J. (2003). The structure and function of complex networks. SIAM Rev., 45:167–256. 10.1137/S003614450342480

[B46] Nicoll R. A. Malenka R. C. (1998). A tale of two transmitters. Science 281, 360–361. 9705712 10.1126/science.281.5375.360

[B47] Orlandi J. G. Stetter O. Soriano J. Geisel T. Battaglia D. (2014). Transfer entropy reconstruction and labeling of neuronal connections from simulated calcium imaging. PLoS ONE 9, e98842. 10.1371/journal.pone.0098842 24905689 PMC4048312

[B48] Platt M. L. Glimcher P. W. (1999). Neural correlates of decision variables in parietal cortex. Nature 400, 233–238. 10421364 10.1038/22268

[B49] Rangan A. V. Cai D. McLaughlin D. W. (2005). Modeling the spatiotemporal cortical activity associated with the line-motion illusion in primary visual cortex. Proc. Natl. Acad. Sci. U.S.A. 102, 18793–18800. 10.1073/pnas.0509481102 16380423 PMC1323193

[B50] Rauch A. La Camera G. Luscher H.-R. Senn W. Fusi S. (2003). Neocortical pyramidal cells respond as integrate-and-fire neurons to in vivo-like input currents. J. Neurophysiol. 90, 1598–1612. 10.1152/jn.00293.2003 12750422

[B51] Renart A. Moreno-Bote R. Wang X. J. Parga N. (2007). Mean-driven and fluctuation-driven persistent activity in recurrent networks. Neural. Comput. 19, 1–46. 10.1162/neco.2007.19.1.1 17134316

[B52] Rosenbaum R. Doiron B. (2014). Balanced networks of spiking neurons with spatially dependent recurrent connections. Phys. Rev. X 4, 021039. 10.1103/PhysRevX.4.021039

[B53] Schmidhuber J. (2015). Deep learning in neural networks: an overview. Neural networks, 61:85–117. 10.1016/j.neunet.2014.09.003 25462637

[B54] Seeley T. D. Visscher P. K. Schlegel T. Hogan P. M. Franks N. R. Marshall J. A. (2012). Stop signals provide cross inhibition in collective decision-making by honeybee swarms. Science 335, 108–111. 10.1126/science.1210361 22157081

[B55] Shadlen M. Newsome W. (1994). Noise, neural codes and cortical organization. Curr. Opin. Neurobiol. 4, 569–579. 7812147 10.1016/0959-4388(94)90059-0

[B56] Shadlen M. N. Newsome W. T. (2001). Neural basis of a perceptual decision in the parietal cortex (area LIP) of the rhesus monkey. J. Neurophysiol. 86, 1916–1936. 10.1152/jn.2001.86.4.1916 11600651

[B57] Shelley M. McLaughlin D. Shapley R. Wielaard J. (2002). States of high conductance in a large-scale model of the visual cortex. J. Comp. Neurosci. 13, 93–109. 10.1023/a:1020158106603 12215724

[B58] Shpiro A. Curtu R. Rinzel J. Rubin N. (2007). Dynamical characteristics common to neuronal competition models. J. Neurophysiol. 97, 462–473. 10.1152/jn.00604.2006 17065254 PMC2702527

[B59] Softky W. R. Koch C. (1993). The highly irregular firing of cortical cells is inconsistent with temporal integration of random EPSPs. J. Neurosci. 13, 334–350. 8423479 10.1523/JNEUROSCI.13-01-00334.1993 PMC6576320

[B60] Sporns O. (2014). Contributions and challenges for network models in cognitive neuroscience. Nat. Neurosci. 17, 652–660. 10.1038/nn.3690 24686784

[B61] Stevenson I. H. Rebesco J. M. Miller L. E. Kording K. P. (2008). Inferring functional connections between neurons. Curr. Opin. Neurobiol. 18, 582–588. 10.1016/j.conb.2008.11.005 19081241 PMC2706692

[B62] Strata P. Harvey R. (1999). Dale's principle. Brain Res. Bull. 50, 349–350. 10643431 10.1016/s0361-9230(99)00100-8

[B63] Svensson E. Apergis-Schoute J. Burnstock G. Nusbaum M. P. Parker D. Schioth H. B. (2018). General principles of neuronal co-transmission: insights from multiple model systems. Front. Neural. Circuits 12, 117. 10.3389/fncir.2018.00117 30728768 PMC6352749

[B64] Troyer T. W. Miller K. D. (1997). Physiological gain leads to high ISI variability in a simple model of a cortical regular spiking cell. Neural Comput. 9, 971–983. 9188190 10.1162/neco.1997.9.5.971

[B65] Tsodyks M. Kenet T. Grinvald A. Arieli A. (1999). Linking spontaneous activity of single cortical neurons and the underlying functional architecture. Science 286, 1943–1946. 10583955 10.1126/science.286.5446.1943

[B66] Usher M. McClelland J. L. (2001). The time course of perceptual choice: the leaky, competing accumulator model. Psychol. Rev. 108, 550–592. 10.1037/0033-295x.108.3.550 11488378

[B67] van Vreeswijk C. Sompolinsky H. (1996). Chaos in neuronal networks with balanced excitatory and inhibitory activity. Science 274, 1724–1726. 8939866 10.1126/science.274.5293.1724

[B68] van Vreeswijk C. Sompolinsky H. (1998). Chaotic balanced state in a model of cortical circuits. Neural Comput. 15, 1321–1371. 9698348 10.1162/089976698300017214

[B69] Vogels T. Abbott L. (2005). Signal propagation and logic gating in networks of integrate-and-fire neurons. J. Neurosci 25, 10786–10795. 10.1523/JNEUROSCI.3508-05.2005 16291952 PMC6725859

[B70] Von Frisch K. (2013). The Dance Language and Orientation of Bees. Cambridge, MA: Harvard University Press.

[B71] Vzquez-Rodrguez B. Surez L. E. Markello R. D. Shafiei G. Paquola C. Hagmann P.. (2019). Gradients of structure-function tethering across neocortex. Proc. Natl. Acad. Sci. U.S.A. 116, 21219–21227. 10.1073/pnas.1903403116 31570622 PMC6800358

[B72] Wang N. Yeung D.-Y. (2013). Learning a deep compact image representation for visual tracking, in Advances in Neural Information Processing Systems (Red Hook, NY: Curran Associates Inc.), 809–817.

[B73] Wang X. J. (2008). Decision making in recurrent neuronal circuits. Neuron 60, 215–234. 10.1016/j.neuron.2008.09.034 18957215 PMC2710297

[B74] Whalley K. (2013). Neural coding: timing is key in the olfactory system. Nat. Rev. Neurosci. 14, 458. 10.1038/nrn3532 23736754

[B75] Yamada W. Koch C. Adams P. (1989). Multiple channels and calcium dynamics, in Methods in Neuronal Modeling: From Synapses To Networks (Cambridge, MA: MIT Press), 97–133.

[B76] Yang S. Gao T. Wang J. Deng B. Lansdell B. Linares-Barranco B. (2021). Efficient spike-driven learning with dendritic event-based processing. Front. Neurosci. 15:97. 10.3389/fnins.2021.601109 33679295 PMC7933681

